# L-3-n-butylphthalide attenuates inflammation response and brain edema in rat intracerebral hemorrhage model

**DOI:** 10.18632/aging.103342

**Published:** 2020-06-21

**Authors:** Zhou Zeng, Xiyu Gong, Zhiping Hu

**Affiliations:** 1 Department of Neurology, The Second Xiangya Hospital of Central South University, Changsha 410011, Hunan, China

**Keywords:** L-3-n-butylphthalide, intracerebral hemorrhage, blood-brain barrier, brain edema, anti-inflammation

## Abstract

L-3-n-butylphthalide(NBP), a compound found in *Apium graveolens* Linn seed extracts, has a therapeutic effect on acute ischemic stroke. The pathological inflammatory pathways and consequent brain edema in intracerebral hemorrhage (ICH) share some similar characteristics with ischemic stroke. We hypothesized that NBP has anti-inflammatory and therapeutic effects on rats with ICH. ICH was induced by an infusion of bacterial collagenase type IV into the unilateral striatum of anesthetized rats. The therapeutic effect of NBP was measured by assessing neurological function, brain water content, blood-brain barrier permeability, and expression of tumor necrosis factor-alpha (TNF-α) and matrix metalloproteinase-9 (MMP-9) around the hematoma 48 hours after surgery. Magnetic resonance imaging was performed 4 and 48 hours after ICH induction, and ICH-induced injured area volumes were measured using T2-weighted images. The NBP treatment group performed better in the neurological function test than the vehicle group. Moreover, in comparison with the vehicle group, NBP group showed a lower expanded hematoma volume, brain water content, blood-brain barrier permeability, and TNF-α/ MMP-9 expression level. Our results indicate that NBP attenuates inflammation and brain edema in rat ICH model. Therefore, our findings also provide a potential therapeutic strategy for the treatment of ICH with NBP.

## INTRODUCTION

Intracerebral hemorrhage (ICH) is the most severe subtype of stroke and is associated with high morbidity and considerable mortality rates in middle- and low-income countries. Despite a continually evolving knowledge of ICH, the mortality rate has not decreased over time [1, 2]. Currently, the 30-day mortality rate of ICH is still as high as 35–50%, with approximately 50% of mortalities occurring within the first two days [3]. Brain injury after ICH is roughly classified into primary and secondary brain injury. Primary brain injury is mainly caused by the destruction and oppression of the brain tissue by the hematoma. Secondary brain injury is mainly due to an inflammatory response, which can aggravate brain edema near the hematoma.

Brain edema after ICH can cause poor outcomes such as severe neurological deficits, herniation, and even death [4]. There are numerous promising preclinical reports on ICH treatment, and most of these are focused on the treatment of primary injuries such as the reduction of intracranial pressure, control of blood pressure, and rehabilitation, and are available clinically. But unfortunately, none of the clinical treatments are centered on alleviating the secondary injury caused by the inflammatory response [5] or increasing the survival rate after ICH [6].

L-3-n-butylphthalide (NBP) is a drug that was originally extracted from the seeds of *Apium graveolens* Linn for acute ischemic cerebrovascular injury. It has been approved by National Medical Products Administration for the treatment of ischemic stroke in China since 2002. Previous studies have shown that NBP could have therapeutic effects on ischemic stroke through multiple mechanisms, including reducing the inflammatory response of ischemic stroke [1], attenuating microglia activation [3], decreasing the levels of cytokines such as tumor necrosis factor-alpha (TNF-α), reducing blood-brain barrier (BBB) damage and brain edema [4], and promoting remyelination process [7].

Although the original cause of the brain damage induced by ICH is different from ischemic stroke, the subsequent inflammatory pathological pathways and consequent brain edema in ICH share many common characteristics with ischemic stroke. Additionally, the secondary ischemic damage often occurs after the initial ICH [8]. Thus, we hypothesized that NBP treatment might have therapeutic effects against ICH. To date, very few studies have focused on the therapeutic effects of NBP on ICH. Here we report the therapeutic effects of NBP on a rat ICH model and explore its underlying molecular mechanisms.

In this study, we investigated the therapeutic effects of NBP on intracerebral hemorrhage in rat model. We found the evidence that NBP ameliorated neurological deficits, decreased hematoma expansion, brain water content, blood-brain barrier permeability and the expression of pro-inflammatory cytokine TNF-α and MMP-9 when dosed following ICH induction. Those results indicate that NBP have an anti-inflammatory effect, and thus may prevent secondary injury in the setting of ICH. Therefore, this study may provide additional prevention and treatment method for intracerebral hemorrhage, and expand the application of NBP.

## RESULTS

### NBP improved the neurological function after ICH

**Figure 1:**
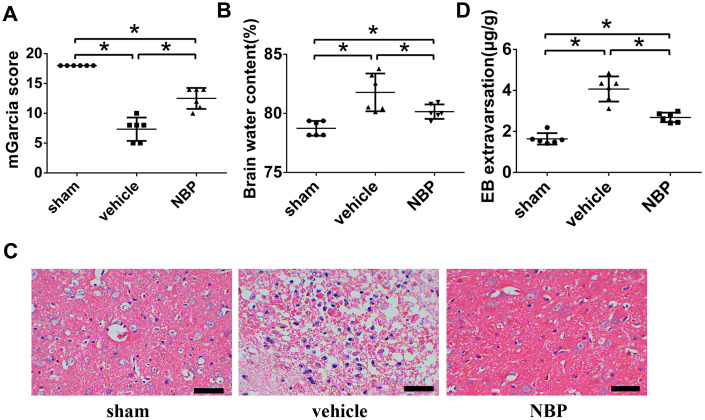
**The therapeutic effects of NBP.** (**A**) NBP improved the neurological function after ICH; (**B**) NBP reduced the brain edema after ICH; (**C**) The HE staining of brain tissues (scale: 200 μm); (**D**) NBP attenuated the BBB permeability after ICH. Data were recorded 48 h after ICH modeling and were presented as the mean ± SD (n = 6, each group). *, P < 0.05. NBP: butylphthalide; BBB: blood-brain barrier; ICH: intracerebral hemorrhage.

One of the consequences of ICH is impaired neurological function. In order to examine the therapeutic effects of NBP after ICH, we first performed the neurological deficit assay using mGarcia neurological scoring system, which has been widely used to investigate early pathophysiological changes. In this scoring system, the more severe the neurological damage, the lower the mGarcia score obtained. At 48h postoperatively, the sham group performed normally according to the modified Garcia score. The rats in vehicle group and NBP group showed a phenomenon of hemiplegia to the left after ICH operation. Although the mGarcia score for vehicle/NBP group were significantly lower than the sham group (P<0.05), which means a significant neurological deficit after ICH, the NBP group’s rats gained a significantly higher mGarcia score than vehicle group (P < 0.05; Figure 1A). These results suggest that NBP treatment can improve and rescue the neurological function after ICH injury effectively.

### NBP reduced the brain edema after ICH

Brain edema is one of the manifestations of brain damage. To explore the effect of NBP on brain edema after ICH, we investigated the extent of brain after saline or NBP treatment. The brain water content in the vehicle group was significantly higher than in the sham group (P < 0.05). In addition, the brain water content in the NBP group was significantly lower than that in the vehicle group (P < 0.05; Figure 1B). Similar results were also observed using hematoxylin eosin (HE) staining. Significant tissue edema was observed in the group vehicle, but NBP treatment remarkably improved brain edema (Figure 1C). These results showed that NBP could reduce the brain edema in ICH model.

### NBP attenuated the BBB permeability after ICH

A modified EB dye method was performed to investigate the vascular permeability of the BBB. The amount of EB exudation in the vehicle group was significantly higher than the amount in the sham group (P < 0.05). Moreover, EB exudation in the NBP group was significantly lower than that in the vehicle group (P < 0.05; Figure 1D). Considering that the brain tissue of the lateral plane of the injured side was approximately 5 mm thick, this result showed that NBP could significantly decrease the EB extravasation significantly in ICH model.

### NBP decreased the ICH-induced injured area

**Figure 2:**
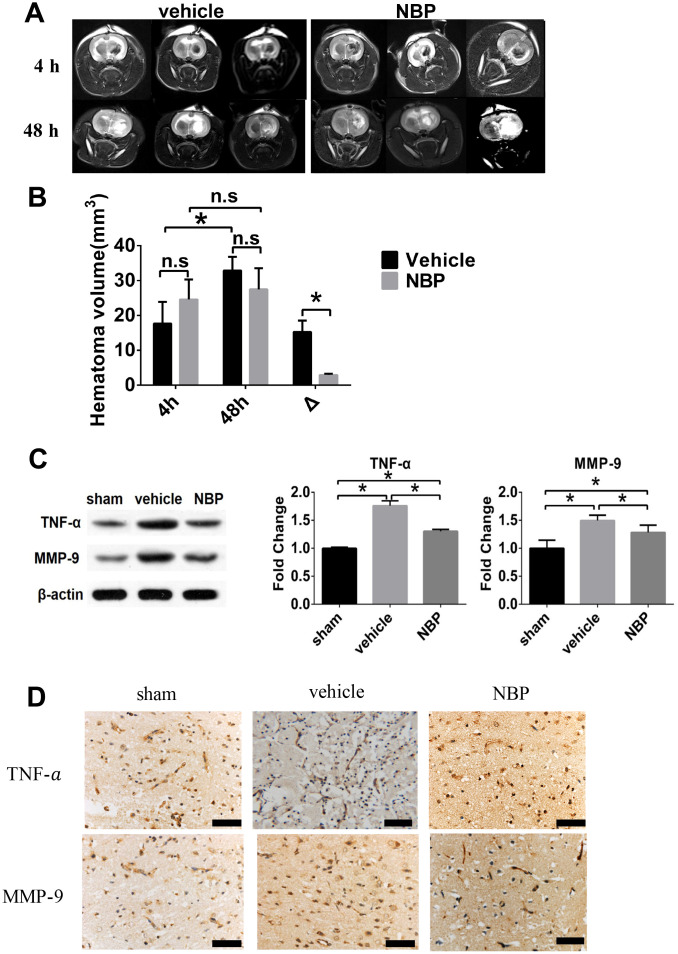
**Effect of NBP on the changes of injured area volume post-ICH.** (**A**) Representative T2-WI images of the vehicle and NBP groups; (**B**) The hematoma volume at different time points (4h and 48h post-ICH) were quantified; (**C**) TNF-α and MMP-9 expression in peri-hematoma brain tissue; (**D**) TNF-α and MMP-9 expression in peri-hematoma brain tissue measured by immunohistochemistry staining (scale: 200 μm). Δ represented the expanded injured area volume, which was calculated by subtracting the volume measured at 48 hours from that at 4 hours. Data are presented as the mean ± SD (n=3, each group). n.s., no significant difference; *, P < 0.05. ICH: intracerebral hemorrhage; NBP: butylphthalide; MMP-9: matrix metalloproteinase-9; TNF-α: tumor necrosis factor-alpha.

In order to visually show the effect of NBP on the expansion of hematoma, we used magnetic resonance imaging (MRI) to observe the brain of ICH model rats. All 6 animals had a clearly and identifiable hematoma develop after ICH insult, and typical T2-weighted images were illustrated in Figure 2. The T2-weighted signal intensity of the injury area maintained hypo-intensity after 4 h, and converted to hyperintensity 48 h after ICH in both the vehicle group and NBP group (Figure 2A). MRI T2WI examination of the head of the rats was performed at 4 h after ICH modeling. It can be seen that the lesion area of the NBP group was approximately the same as that of the vehicle group, and there was no statistically significant difference (p >0.05). MRI T2WI was performed again at 48 hours after surgery. During the period from 4 hours to 48 hours after surgery, the lesion area of the vehicle group increased significantly, while the lesion area of the NBP group showed no significant change. That is, the expansive lesion area of the NBP group was significantly smaller than that of the vehicle group (15.22 ± 3.31 mm^3^ vs. 2.91 ± 0.40 mm^3^, P < 0.05; Figure 2B), indicating that NBP can effectively inhibit cerebral edema caused by ICH.

### NBP inhibited the expression of pro-inflammatory factor TNF-α and MMP-9

Previous studies indicated that inflammation could be the key mechanism of edema formation after ICH [9]. To explore whether NBP has anti-inflammatory effect in ICH rat model, we first examined the expression level of pro-inflammatory factor TNF-α in the brain tissue surrounding the hematoma area. The western blotting results showed that ICH injury led to an increase expression of TNF-α. Meanwhile, the NBP treatment can significantly reduce the expression of TNF-α compared with vehicle group. We also examined the expression of MMP-9, a downstream of TNF-α in the peri-hematoma cortex tissue. In the vehicle group, the upregulated TNF-α resulted in an upregulation of MMP-9 compared with sham group. In addition, the expression level of MMP-9 in the NBP group fell down compared with vehicle group (Figure 2C). Similar findings related with the expression of TNF-α and MMP-9 was observed using immunohistochemistry staining (Figure 2D). These results indicated that NBP have an anti-inflammatory effect, and thus may prevent secondary injury after ICH.

### NBP inhibited oxidative stress and DNA damage

**Figure 3:**
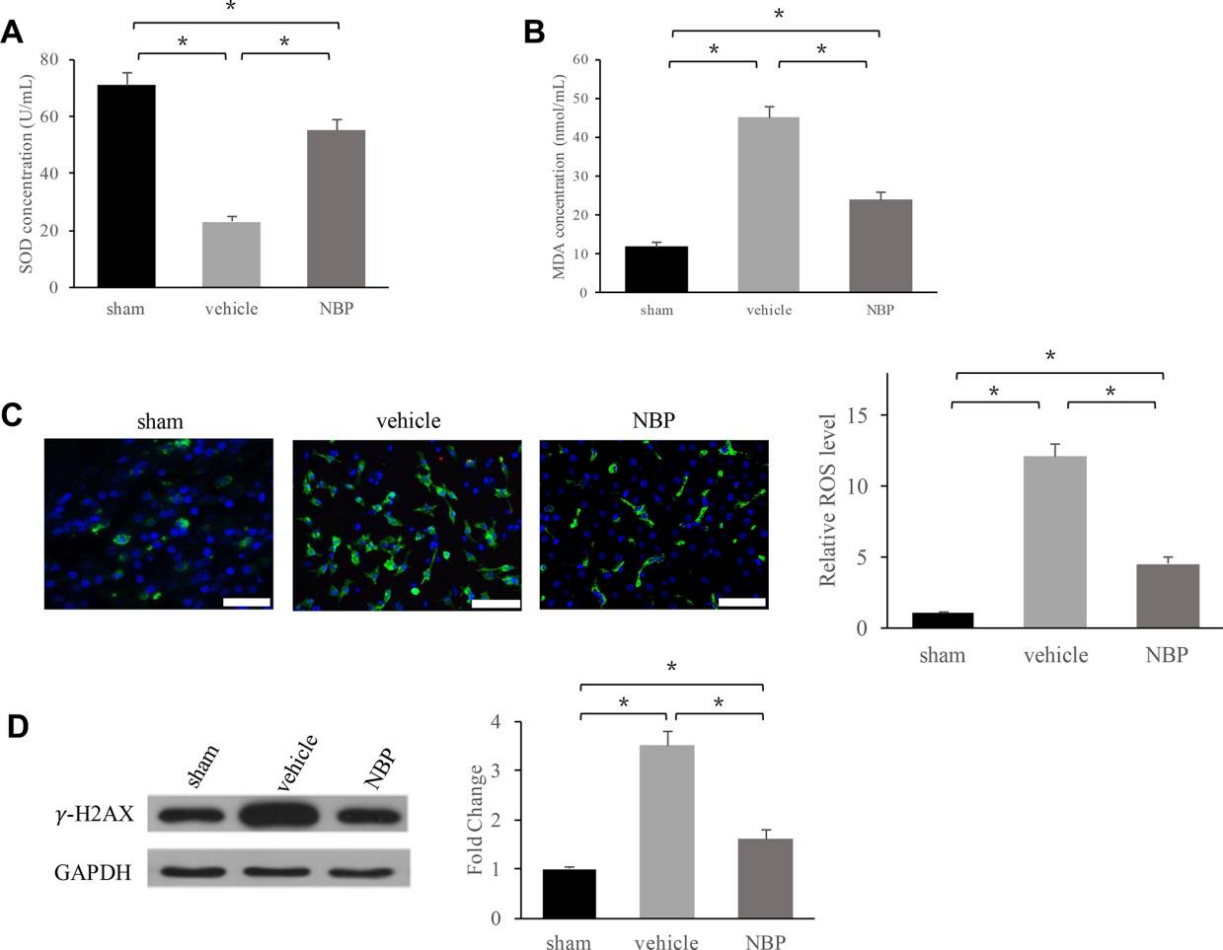
**NBP inhibited oxidative stress and DNA damage:** (**A**) SOD level in the serum; (**B**) MDA level in the serum; (**C**) ROS level in the brain tissues (scale: 300 μm); (**D**) γ-H2AX expression in peri-hematoma brain tissue. Data are presented as the mean ± SD (n=3, each group). *, P < 0.05. ROS: reactive oxygen species; SOD: Super oxide dismutase; MDA: Malondialdehyde.

Oxidative stress has been considered to be closely linked with inflammation, so we investigated the influence of NBP on super oxide dismutase (SOD), malondialdehyde (MDA), reactive oxygen species (ROS) levels. We found that in the group vehicle SOD (Figure 3A) was decreased, but MDA (Figure 3B) and ROS (Figure 3C) increased significantly. However, NBP treatment remarkably reversed these trends and inhibited oxidative stress. Meanwhile, DNA damage marker, γ-H2AX, was measured after NBP treatment. We found that NBP remarkably suppressed the level of γ-H2AX suggesting that NBP could inhibit DNA damage (Figure 3D).

## DISCUSSION

ICH is a stroke type with a very high mortality rate. At the beginning, tissue damage activated local inflammation, then the BBB was destroyed, followed by the recruitment of circulating inflammatory cells and subsequent secondary immunopathological changes, and finally the response of tissue repair [10]. Preclinical data suggested that elimination of initial neuro inflammation should be a key step in the treatment of ICH injury. For a long time, researchers have been looking for drugs to reduce inflammation and edema after intracerebral hemorrhage. A recent study demonstrated that activator protein 1 inhibitor SR11302 could reduce the expression of microglial IL-6 and TNF-α and brain-infiltrating leukocytes and thus attenuating inflammation and edema in ICH mice [11]. Our study demonstrates that treatment with NBP can partially relieve the symptoms of intracerebral hemorrhage, including the improvement of neurological outcomes, reduction of brain edema, attenuation of the BBB permeability, also decrease of ICH-induced injured area. The mechanism is to suppress the inflammatory reaction after ICH. The findings of our investigation provide new evidence that NBP may be effective in the treatment of ICH in addition to confirming the previously hypothesized therapeutic effect of NBP on ischemic stroke. To date, there have been few reports on the evaluation of NBP in an ICH model. We firstly proved that NBP can reduce inflammation and brain edema in ICH rats.

**Figure 4:**
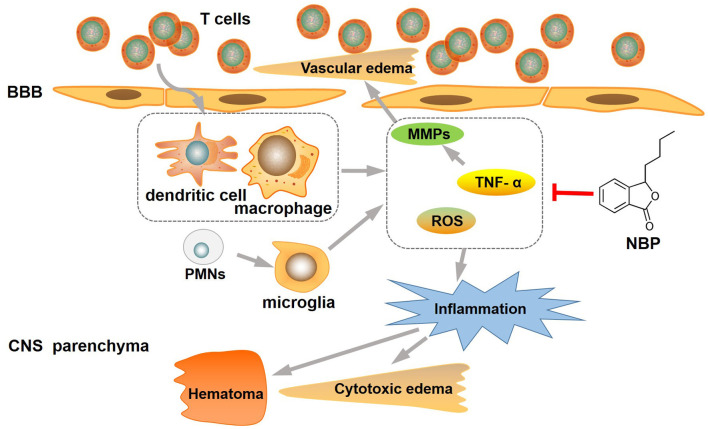
**NBP attenuated inflammation and brain edema in intracerebral hemorrhage.** NBP treatment substantially inhibit the inflammation, reduced ICH-induced brain edema, and then protected the BBB from disruption. BBB: blood-brain barrier; CNS: central nervous system; MMP-9: matrix metalloproteinase-9; NBP: butylphthalide; ROS:reactive oxygen species; TNF-α: tumor necrosis factor-alpha.

Brain edema plays an important role in secondary brain injury after ICH [12]. Accumulating data from preclinical and clinical studies suggest that inflammation could be the key mechanism of edema formation after ICH, causing cell swelling and BBB disruption. Previous studies have demonstrated that the inflammatory response could take place around the hematoma, with an infiltration of neutrophils, macrophages, and activated microglia [13]. Microglia mediated neuro inflammation plays an important role in the inflammatory injury of intracerebral hemorrhage [14, 15]. It has been reported that other chemical compounds like minocycline, curcumin, and magnolol, can also reduce microglia mediated neuro inflammation in ICH animal models, showing the potential application value in the treatment of intracranial hemorrhage. [9, 15, 16]. Neutrophils or polymorphonuclear leukocytes (PMNs) are the first leukocytes to infiltrate the nervous system after ICH, and could cause direct neurotoxicity to brain tissue and cell swelling by releasing TNF-α, matrix metalloproteinases (MMPs), and other cytokines [4, 17]. TNF-α is known for its ability to cause inflammatory reactions. TNF-α appears to be involved in inflammation, BBB, thrombogenesis, and vascular changes associated with brain injury. TNF-α has been shown to upregulate MMP expression, especially MMP-9, in inflammatory reactions. MMP-9 is one of the most important MMPs associated with BBB damage and could lead to an increase in cerebral vessel permeability [18, 19]. MMPs might also cause further damage by stimulating microglia, which could also be activated by thrombin and heme after ICH [20–22]. The main purpose of microglia cell activation is to clear the hematoma, but excessive activation can also result in neurotoxicity to brain tissue by releasing diverse toxic factors such as cytokines, chemokines, proteases, ROS, and heme oxygenase [21, 23]. In brief, intracerebral hemorrhage can cause inflammatory reactions by activating PMNs, microglia, and other cells, which can upregulate the expression of TNF-α, MMP-9, and other inflammatory cytokines, thereby causing BBB destruction and cell swelling and aggravating cerebral edema (Figure 4).

Despite numerous promising preclinical studies on ICH, only a few experimental approaches are aimed at reducing edema after ICH, and these treatments are limited in terms of their clinical utility [24–30]. Previous studies have shown that NBP has an obvious therapeutic effect on acute ischemic stroke by alleviating the inflammatory response, reducing brain edema [18, 31, 32]. Therefore, to assess the overlapping inflammatory and brain edema pathological mechanisms in ischemic and hemorrhagic stroke, we used an intracerebral hemorrhage model and explored the therapeutic effect of NBP in rats with intracerebral hemorrhage.

Our data showed that treatment with NBP reduced the brain water content after ICH. Consistent with the reduction in cerebral edema, the BBB permeability was also reduced. These data suggest that NBP could reduce brain edema and alleviate BBB disruption after ICH. The T2-weighted images showed that the expanded hematoma volume, which is one of the most important factor enhancing brain injury after ICH [19, 33], was significantly reduced in the NBP group. These images indicate that treatment with NBP post-ICH did not increase the risk of re-bleeding or hematoma enlargement, and might even reduce such risks after ICH.

Compared with the vehicle group, the initial (4h) hemorrhage volume of the NBP group appeared a little bit larger than that of the vehicle group, but there was no statistically significant difference. Previous study showed that NBP has antiplatelet effects, and antiplatelet medicines may increase the risk of intracerebral hemorrhage [34–36]. This was consistent with our results. Moreover, increasing the number of test animals can eliminate the influence of individual differences. However, 48 hours later, the volume of the hemorrhage in the NBP group became a little bit smaller than that in the vehicle group but without statistically significant difference. It was worthwhile pointing out that the enlarged hemorrhage became smaller in the 4-48h period after NBP treatment. In NBP group, the volume of cerebral hemorrhage at 48h was not significantly different from that at 4h, while in the vehicle group, the volume of hemorrhage at 48h was significantly expanded. The aggravation of clinical symptoms of intracerebral hemorrhage is related to the expansion of hemorrhagic foci and cerebral edema, and this re-expansion occurs mostly in the early stage [33, 37]. Therefore, the decrease of hematoma expansion after NBP treatment also shows the safety of NBP in ICH treatment.

Antihypertensive Treatment of Acute Cerebral Hemorrhage 2 (ATACH-2) trial found that anti-hypertensive treatment could reduce hematoma expansion, but had no significant effect on outcome of ICH [38]. Early hematoma enlargement is still an important cause of aggravation and poor prognosis of cerebral hemorrhage [33]. The ATACH-2 trial did not show that the benefit of hypotensive therapy may be related to over hypotension. In addition to the re-expansion of cerebral hemorrhage volume, there may be other factors such as initial lesion size or ICH localization play key roles in recovery. In clinical trials, treatment such as antiplatelet or anticoagulant therapy will affect the outcome of ICH [39]. In our animal model, neurological function tests indicated that NBP contributes to the partial recovery of brain function after ICH injury.

Meanwhile, NBP could inhibit the expression of both TNF-α and MMP-9 protein content around the hematoma, which play key roles in the mechanism of the inflammatory response and edema formation after ICH. The results of this study showed the underlying molecular mechanism of NBP in ICH rats via an inhibitory action on inflammation. A recent study also confirmed that NBP had an anti-inflammatory properties and thus promote the survival of oligodendrocytes in a mouse cuprizone demyelination model [40]. Decreasing the expression of TNF-α and other inflammatory cytokines could reduce cell swelling and cytotoxic edema after ICH. In addition, reducing the activation of MMP-9 could alleviate the damage to BBB permeability and vasogenic edema after ICH.

Our research provides the potential application value of NBP. However, there are still some limitations in this study. First, our results demonstrated that NBP could contribute to short term recovery of brain functions at least. A study involving 536 patients with acute cerebral hemorrhage showed that the early stage of cerebral hemorrhage was aggravated by neurological deficit. About 83% of patients have hematoma enlargement in early stage [41]. Clinically, the majority of hematoma enlargement in the early stage of cerebral hemorrhage occurred within 3-24 hours [37]. We investigated the early therapeutic effects of NBP in ICH injury. However, as mentioned above, ICH response lasts for 72 hours or even longer [10]. The extension of investigating period to 72 hours or longer time periods post ICH will help us better track the efficacy of NBP. Second, a previous study showed that after a single oral administration of 100 mg/kg NBP, the highest inhibition rate of NBP on platelets was 56% in the first 2 hours in normal rats [42]. Considering the inhibition of NBP on platelets, we only used a safe low dose of NBP in this investigation. In the follow-up experiment, we will use multiple doses to see whether NBP is dose-dependent on the treatment of ICH. Third, female animals shall be utilized in future studies to address any sex-related differences that may exist. In addition, the therapeutic effects of NBP, which might involve other mechanisms in addition to anti-inflammation, are not fully understood. Therefore, for the future treatment of ICH, further animal studies that focus on the molecular mechanism by selective blockade of the ICH relative pathway are necessary. Finally, previous studies showed that NBP could significantly inhibit platelet activation and might be an effective antiplatelet drug for ischemic stroke [34–36]. Therefore, future preclinical and translational studies are also needed to address the safety effects of NBP for the treatment ICH.

In summary, our results demonstrate that NBP inhibits the expression of TNF-α and MMP-9, thereby reducing inflammatory reactions, BBB damage, the ICH-induced injured area, and both cytotoxic and vasogenic edema. Our findings provide evidence for the treatment of intracerebral hemorrhage and the clinical application of NBP.

## MATERIALS AND METHODS

### Experimental animals

A total of 45 pathogen-free and healthy adult male Sprague-Dawley rats (10 weeks old, 280-320 g) were purchased from Silikejingda Experimental Animals Corporation (Changsha, China). All experimental procedures on animals were conducted in accordance with the Guidelines for the Care and Use of Animals issued by the International Guidance Center of Life [43] and approved by the Animal Care and Use Committee of Central south University (Approved number: 2019-042). The rats were housed in the Hunan Provincial People’s Hospital, China, in a controlled environment at a temperature of 22 ± 2 °C and a humidity of 40–50%, with a 12 h dark/ 12 h light cycle. Water and food were provided ad libitum. The rats were given 1 week to adjust to the new environment before the start of the experiment, then were randomly divided into three groups: (1) sham group (n = 15) without ICH modeling; (2) vehicle group (n = 15) with ICH modeling and saline treatment; (3) NBP group (n = 15) with ICH modeling and NBP treatment. The chemical structure of NBP and schematic diagram of experimental design were shown in the Supplementary Figure 1. In subsequent experiments, 6 or 3 rats were used in each group. The exact number of animals was indicated in the figure legends.

### Establishment of ICH models and drug administration

After the rats were anesthetized by intramuscular injection with ketamine (60 mg/kg) and xylazine (6 mg/kg), 2 μL (0.2 μL/min) of saline containing 0.4 units of bacterial collagenase (type IV; Sigma-Aldrich, Germany) were infused via a 30-gauge needle into the striatum (1.0 mm posterior, 4.0 mm lateral, 6.0 mm ventral to the cortical surface) [44]. The sham group was infused with 2 μL of saline. The needle was left in place for an additional 10 minutes following infusion to prevent backflow. The craniotomies were sealed with bone wax. The rats were left to recover in individual cages in the animal center under a 12/12 h light/dark cycle with free access to food and water. Rats in the NBP group were treated with NBP sodium chloride 30 minutes after ICH induction (CSPC NBP Pharmaceutical Co., Ltd., China, 25 mg/kg, twice per day, intraperitoneally [18, 31]), while rats in the sham and vehicle groups were given the same amount of normal saline by intraperitoneal injection.

### Evaluation of neurological function

An investigator blinded to the treatment scheme conducted a modified Garcia test (mGarcia) on the experimental rats before ICH (Day 0) and 48 h after treatment. Neurological function was graded on a scale of 0 -18 (normal score: 18; maximum deficit score: 0). The mGarcia is a composite test of motor, sensory, and balance functions [8]. Rats with an abnormal score (< 18) before ICH were excluded from the experiment.

### Measurement of brain water content

The rats were deeply anesthetized by intramuscular injection with ketamine (60 mg/kg) and xylazine (6 mg/kg) and decapitated. The brain water content was measured using a drying method [45]. With the cerebellar tissue removed, the wet weight of the right and left hemispheres was measured. Wet weight was measured using an MA110 electronic analytical balance (Shanghai Second Balance Instrument Factory, Shanghai, China). The brain tissues were then placed in an oven at 110 °C and dried for 24 h. The left and right hemispheres were then measured for dry weight. Brain water content was calculated using the Elliot formula: brain water content (%) = (wet weight - dry weight)/wet weight × 100%.

### Assessment of BBB permeability

The vascular permeability of the BBB was evaluated using a modified Evans blue dye method [46]. Briefly, EB (2% in 0.9% saline; 3 mL/kg) was administered intravenously 2 hours prior to sacrifice. Via a thoracotomy performed under chloral hydrate anesthesia, intracardiac perfusion was performed through the left ventricle with saline to remove the intravascular EB dye and continued until the fluid from the right atrium became colorless. The rats were then decapitated and the brains quickly removed. The brain was weighed and homogenized in 2 mL of a 50% trichloroacetic acid solution. After centrifugation at 10,000 g for 20 min, the supernatants were diluted with ethanol (1:3), and the EB concentration was determined using a spectrophotometer at 620 nm for absorbance against a standard curve. EB extravasation was expressed in μg/g.

### MRI examination of the ICH-induced injured area

Serial MRI examination was performed using a 3.0 T MRI (MAGNETOM Skyra MR D13, SIEMENSAGFWB: SIE, Germany) with a 4.3 cm diameter surface coil to assess the hematoma expansion 4 and 48 h after ICH induction (total n = 6, vehicle and NBP group). During the MRI examination, rats were anesthetized and placed in a prone position using the same method used for ICH induction. The MRI protocol consisted of a T2-weighted 3-dimension fast recovery fast spin echo (FRFSE) sequence with the following relevant parameters: repetition time (TR)/echo time (TE): 5860/119 ms, matrix size = 256 × 256, field of view (FOV) = 162 mm × 100 mm, slice thickness = 2 mm. Every image was converted to and saved as a Digital image and communications in medicine file, and a blinded observer measured the injured area from the T2-weighted images using Image J software (version 1.52, National Institutes of Health, USA). The injured area contour was extracted by manually outlining the regions of hyperintensity/hypointensity that were distinct from the surrounding brain tissue in each image [47]. The ICH-induced injured volume was calculated by Coniglobus formula. Volume = π/6 × length × width × layer thickness × layer number. The expanded injured area volume was calculated by subtracting the volume measured at 48 hours from that at 4 hours.

### Western blotting

Forty-eight hours after ICH, three rats were randomly selected from each group. The rats received deep anesthesia followed by perfusion with 4% paraformaldehyde, and then were decapitated. Then, protein lysis and western blotting were conducted as described [48, 49]. The protein in the peri-hematoma brain tissue was extracted using a cell lysate, and the protein content in each slice was determined using Coomassie brilliant blue staining. Protein samples (50–100 μg) were loaded onto an 10% sodium dodecyl sulfate polyacrylamide gel electrophoresis, transferred to nitrocellulose film, blocked using 5% skim milk powder, and subsequently incubated with primary antibodies diluted in blocking solution overnight. The following antibodies were used for western blot: mouse monoclonal anti-TNF-α (1:500, Proteintech Biotechnology, Rosemont, California), mouse monoclonal anti-MMP-9 (1:200, Proteintech Biotechnology), and mouse monoclonal anti-β-actin (1:4000, Proteintech Biotechnology). After washing with tris-buffered saline (TBS) 3 times, the membrane was incubated with secondary antibodies (horseradish peroxidase-conjugated anti-mouse or anti-goat IgG, 1:3000, Proteintech Biotechnology) at room temperature of 22 ± 2 °C for 1 h. Slices were developed using an enhanced chemiluminescence detection reagents for 3 min, exposed, and then fixed. The mean absorbance value from the western blot analysis was analyzed using a digital imaging analysis system, with the target absorbance serving as the reference.

### HE staining

HE staining was performed as described previously [50, 51]. Briefly, brain tissues were isolated after the sacrifice of mice, and then fixed with 10% formalin for 48 h. OCT compound (Sigma, USA) was applied for tissue embedding, and 8-μm thickness sections were made using a frozen microtome. Five slides in each group were used for HE staining. Zeiss AxioVision (Jena, Germany) was applied for capturing.

### Immunohistochemistry staining

Immunohistochemistry staining was conducted as described previously [52]. Briefly, tissues were fixed with 10% formalin for 48 h. Tissue embedding using OCR compound and sections were conducted. The slides were heated for antigen repair, and washed twice (5 min/time). 5% H_2_O_2_ was used to culture tissues for 15 min, and then slides were washed twice (5 min/time). After blocking with 5% goat serum, tissues were incubated with primary antibodies at 񀂰C overnight. After washing twice (5 min/time), secondary antibody was used for incubation for 2 h at room temperature. Then DAB regent was used to incubate tissues, and Zeiss AxioVision (Jena, Germany) was used for capturing.

### Statistical analysis

Data analysis was performed using GraphPad Prism, version 6.01 (GraphPad Software Inc., San Diego, CA, USA). All results are expressed as mean ± SD. Group differences were assessed using Student’s t test. P < 0.05 was used to indicate a significant difference.

## Supplementary Material

Supplementary Figure 1

## References

[1] Flower O, Smith M. The acute management of intracerebral hemorrhage. Curr Opin Crit Care. 2011; 17:106–14. 10.1097/MCC.0b013e328342f823 21169826

[2] Bashir MU, Bhagra A, Kapa S, McLeod CJ. Modulation of the autonomic nervous system through mind and body practices as a treatment for atrial fibrillation. Rev Cardiovasc Med. 2019; 20:129–37. 10.31083/j.rcm.2019.03.517 31601087

[3] Zahuranec DB, Gonzales NR, Brown DL, Lisabeth LD, Longwell PJ, Eden SV, Smith MA, Garcia NM, Hoff JT, Morgenstern LB. Presentation of intracerebral haemorrhage in a community. J Neurol Neurosurg Psychiatry. 2006; 77:340–44. 10.1136/jnnp.2005.077164 16484640 PMC2077701

[4] Zheng H, Chen C, Zhang J, Hu Z. Mechanism and therapy of brain edema after intracerebral hemorrhage. Cerebrovasc Dis. 2016; 42:155–69. 10.1159/000445170 27110940

[5] Keep RF, Hua Y, Xi G. Intracerebral haemorrhage: mechanisms of injury and therapeutic targets. Lancet Neurol. 2012; 11:720–31. 10.1016/S1474-4422(12)70104-7 22698888 PMC3884550

[6] Pouratian N, Kassell NF, Dumont AS. Update on management of intracerebral hemorrhage. Neurosurg Focus. 2003; 15:E2. 10.3171/foc.2003.15.4.2 15344895

[7] Cheng X, Wang H, Liu C, Zhong S, Niu X, Zhang X, Qi R, Zhao S, Zhang X, Qu H, Zhao C. Dl-3-n-butylphthalide promotes remyelination process in cerebral white matter in rats subjected to ischemic stroke. Brain Res. 2019; 1717:167–75. 10.1016/j.brainres.2019.03.017 30986406

[8] Garcia JH, Wagner S, Liu KF, Hu XJ. Neurological deficit and extent of neuronal necrosis attributable to middle cerebral artery occlusion in rats. Statistical validation. Stroke. 1995; 26:627–34. 10.1161/01.str.26.4.627 7709410

[9] Zhou F, Jiang Z, Yang B, Hu Z. Magnolol exhibits anti-inflammatory and neuroprotective effects in a rat model of intracerebral haemorrhage. Brain Behav Immun. 2019; 77:161–67. 10.1016/j.bbi.2018.12.018 30597199

[10] Askenase MH, Sansing LH. Stages of the inflammatory response in pathology and tissue repair after intracerebral hemorrhage. Semin Neurol. 2016; 36:288–97. 10.1055/s-0036-1582132 27214704 PMC4956485

[11] Wei CJ, Li YL, Zhu ZL, Jia DM, Fan ML, Li T, Wang XJ, Li ZG, Ma HS. Inhibition of activator protein 1 attenuates neuroinflammation and brain injury after experimental intracerebral hemorrhage. CNS Neurosci Ther. 2019; 25:1182–88. 10.1111/cns.13206 31392841 PMC6776742

[12] Xi G, Keep RF, Hoff JT. Mechanisms of brain injury after intracerebral haemorrhage. Lancet Neurol. 2006; 5:53–63. 10.1016/S1474-4422(05)70283-0 16361023

[13] Gong C, Hoff JT, Keep RF. Acute inflammatory reaction following experimental intracerebral hemorrhage in rat. Brain Res. 2000; 871:57–65. 10.1016/s0006-8993(00)02427-6 10882783

[14] Lan X, Han X, Li Q, Yang QW, Wang J. Modulators of microglial activation and polarization after intracerebral haemorrhage. Nat Rev Neurol. 2017; 13:420–33. 10.1038/nrneurol.2017.69 28524175 PMC5575938

[15] Yang Z, Zhao T, Zou Y, Zhang JH, Feng H. Curcumin inhibits microglia inflammation and confers neuroprotection in intracerebral hemorrhage. Immunol Lett. 2014; 160:89–95. 10.1016/j.imlet.2014.03.005 24680995

[16] Yang H, Gao XJ, Li YJ, Su JB, E TZ, Zhang X, Ni W, Gu YX. Minocycline reduces intracerebral hemorrhage-induced white matter injury in piglets. CNS Neurosci Ther. 2019; 25:1195–206. 10.1111/cns.13220 31556245 PMC6776747

[17] Nguyen HX, O’Barr TJ, Anderson AJ. Polymorphonuclear leukocytes promote neurotoxicity through release of matrix metalloproteinases, reactive oxygen species, and TNF-alpha. J Neurochem. 2007; 102:900–12. 10.1111/j.1471-4159.2007.04643.x 17561941

[18] Xu HL, Feng YP. Inhibitory effects of chiral 3-n-butylphthalide on inflammation following focal ischemic brain injury in rats. Acta Pharmacol Sin. 2000; 21:433–38. 11324442

[19] Sorokin L. The impact of the extracellular matrix on inflammation. Nat Rev Immunol. 2010; 10:712–23. 10.1038/nri2852 20865019

[20] Chen S, Zeng L, Hu Z. Progressing haemorrhagic stroke: categories, causes, mechanisms and managements. J Neurol. 2014; 261:2061–78. 10.1007/s00415-014-7291-1 24595959 PMC4221651

[21] van Rossum D, Hanisch UK. Microglia. Metab Brain Dis. 2004; 19:393–411. 10.1023/b:mebr.0000043984.73063.d8 15554430

[22] Lin S, Yin Q, Zhong Q, Lv FL, Zhou Y, Li JQ, Wang JZ, Su BY, Yang QW. Heme activates TLR4-mediated inflammatory injury via MyD88/TRIF signaling pathway in intracerebral hemorrhage. J Neuroinflammation. 2012; 9:46. 10.1186/1742-2094-9-46 22394415 PMC3344687

[23] Taylor RA, Sansing LH. Microglial responses after ischemic stroke and intracerebral hemorrhage. Clin Dev Immunol. 2013; 2013:746068. 10.1155/2013/746068 24223607 PMC3810327

[24] Kitaoka T, Hua Y, Xi G, Nagao S, Hoff JT, Keep RF. Effect of delayed argatroban treatment on intracerebral hemorrhage-induced edema in the rat. Acta Neurochir Suppl. 2003; 86:457–61. 10.1007/978-3-7091-0651-8_94 14753486

[25] Lee ST, Chu K, Jung KH, Kim J, Kim EH, Kim SJ, Sinn DI, Ko SY, Kim M, Roh JK. Memantine reduces hematoma expansion in experimental intracerebral hemorrhage, resulting in functional improvement. J Cereb Blood Flow Metab. 2006; 26:536–44. 10.1038/sj.jcbfm.9600213 16107786

[26] Lee ST, Chu K, Sinn DI, Jung KH, Kim EH, Kim SJ, Kim JM, Ko SY, Kim M, Roh JK. Erythropoietin reduces perihematomal inflammation and cell death with eNOS and STAT3 activations in experimental intracerebral hemorrhage. J Neurochem. 2006; 96:1728–39. 10.1111/j.1471-4159.2006.03697.x 16539688

[27] Nakamura T, Keep RF, Hua Y, Park JW, Itano T, Nagao S, Hoff JT, Xi GH. Intracerebral hemorrhage induces edema and oxidative stress and alters N-methyl-D-aspartate receptor subunits expression. Acta Neurochir Suppl. 2005; 95:421–424. 10.1007/3-211-32318-X_86 16463894

[28] Wagner KR, Hua Y, de Courten-Myers GM, Broderick JP, Nishimura RN, Lu SY, Dwyer BE. Tin-mesoporphyrin, a potent heme oxygenase inhibitor, for treatment of intracerebral hemorrhage: in vivo and in vitro studies. Cell Mol Biol (Noisy-le-grand). 2000; 46:597–608. 10872746

[29] Wu G, Huang FP. Effects of venom defibrase on brain edema after intracerebral hemorrhage in rats. Acta Neurochir Suppl. 2005; 95:381–87. 10.1007/3-211-32318-x_78 16463886

[30] Liew HK, Hsu CW, Wang MJ, Kuo JS, Li TY, Peng HF, Wang JY, Pang CY. Therapeutic benefit of urocortin in rats with intracerebral hemorrhage. J Neurosurg. 2012; 116:193–200. 10.3171/2011.8.JNS101637 21981644

[31] Hu J, Wen Q, Wu Y, Li B, Gao P. The effect of butylphthalide on the brain edema, blood-brain barrier of rats after focal cerebral infarction and the expression of rho a. Cell Biochem Biophys. 2014; 69:363–68. 10.1007/s12013-013-9808-0 24442989

[32] Zhao CY, Lei H, Zhang Y, Li L, Xu SF, Cai J, Li PP, Wang L, Wang XL, Peng Y. L-3-n-butylphthalide attenuates neuroinflammatory responses by downregulating JNK activation and upregulating heme oxygenase-1 in lipopolysaccharide-treated mice. J Asian Nat Prod Res. 2016; 18:289–302. 10.1080/10286020.2015.1099524 26675131

[33] Hemphill JC 3rd, Greenberg SM, Anderson CS, Becker K, Bendok BR, Cushman M, Fung GL, Goldstein JN, Macdonald RL, Mitchell PH, Scott PA, Selim MH, Woo D, et al. Guidelines for the Management of Spontaneous Intracerebral Hemorrhage: A Guideline for Healthcare Professionals From the American Heart Association/American Stroke Association. Stroke. 2015; 46:2032–2060. 10.1161/STR.0000000000000069 26022637

[34] Ye J, Zhai L, Zhang S, Zhang Y, Chen L, Hu L, Zhang S, Ding Z. DL-3-n-butylphthalide inhibits platelet activation via inhibition of cPLA2-mediated TXA2 synthesis and phosphodiesterase. Platelets. 2015; 26:736–44. 10.3109/09537104.2014.989826 25734213

[35] Wang XL, Wang ZY, Ling JJ, Zhang YH, Yin J. Synthesis and biological evaluation of nitric oxide (NO)-hydrogen sulfide (H_2_S) releasing derivatives of (S)-3-n-butylphthalide as potential antiplatelet agents. Chin J Nat Med. 2016; 14:946–53. 10.1016/S1875-5364(17)30021-3 28262123

[36] Ma F, Gao Y, Qiao H, Hu X, Chang J. Antiplatelet activity of 3-butyl-6-bromo-1(3H)-isobenzofuranone on rat platelet aggregation. J Thromb Thrombolysis. 2012; 33:64–73. 10.1007/s11239-011-0647-9 22057435

[37] Davis SM, Broderick J, Hennerici M, Brun NC, Diringer MN, Mayer SA, Begtrup K, Steiner T, and Recombinant Activated Factor VII Intracerebral Hemorrhage Trial Investigators. Hematoma growth is a determinant of mortality and poor outcome after intracerebral hemorrhage. Neurology. 2006; 66:1175–81. 10.1212/01.wnl.0000208408.98482.99 16636233

[38] Qureshi AI, Palesch YY, Barsan WG, Hanley DF, Hsu CY, Martin RL, Moy CS, Silbergleit R, Steiner T, Suarez JI, Toyoda K, Wang Y, Yamamoto H, Yoon BW, and ATACH-2 Trial Investigators and the Neurological Emergency Treatment Trials Network. Intensive blood-pressure lowering in patients with acute cerebral hemorrhage. N Engl J Med. 2016; 375:1033–43. 10.1056/NEJMoa1603460 27276234 PMC5345109

[39] Al-Shahi Salman R, Frantzias J, Lee RJ, Lyden PD, Battey TW, Ayres AM, Goldstein JN, Mayer SA, Steiner T, Wang X, Arima H, Hasegawa H, Oishi M, et al, VISTA-ICH Collaboration, and ICH Growth Individual Patient Data Meta-analysis Collaborators. Absolute risk and predictors of the growth of acute spontaneous intracerebral haemorrhage: a systematic review and meta-analysis of individual patient data. Lancet Neurol. 2018; 17:885–94. 10.1016/S1474-4422(18)30253-9 30120039 PMC6143589

[40] Wu Y, Dong L, Huang Q, Sun L, Liao Y, Tang Y, Wu Y. Multiple functional therapeutic effects of DL-3-n-butylphthalide in the cuprizone model of demyelination. Life Sci. 2019; 232:116501. 10.1016/j.lfs.2019.05.057 31163175

[41] Fan JS, Chen YC, Huang HH, How CK, Yen DH, Huang MS. The association between on-scene blood pressure and early neurological deterioration in patients with spontaneous intracerebral haemorrhage. Emerg Med J. 2015; 32:239–43. 10.1136/emermed-2013-203114 24123169

[42] Peng Y, Zeng X, Feng Y, Wang X. Antiplatelet and antithrombotic activity of L-3-n-butylphthalide in rats. J Cardiovasc Pharmacol. 2004; 43:876–81. 10.1097/00005344-200406000-00018 15167282

[43] Demers G, Griffin G, De Vroey G, Haywood JR, Zurlo J, Bédard M. Animal research. Harmonization of animal care and use guidance. Science. 2006; 312:700–01. 10.1126/science.1124036 16675685

[44] MacLellan CL, Silasi G, Poon CC, Edmundson CL, Buist R, Peeling J, Colbourne F. Intracerebral hemorrhage models in rat: comparing collagenase to blood infusion. J Cereb Blood Flow Metab. 2008; 28:516–25. 10.1038/sj.jcbfm.9600548 17726491

[45] Wang T, Chen X, Wang Z, Zhang M, Meng H, Gao Y, Luo B, Tao L, Chen Y. Poloxamer-188 can attenuate blood-brain barrier damage to exert neuroprotective effect in mice intracerebral hemorrhage model. J Mol Neurosci. 2015; 55:240–50. 10.1007/s12031-014-0313-8 24770901

[46] Esen F, Erdem T, Aktan D, Orhan M, Kaya M, Eraksoy H, Cakar N, Telci L. Effect of magnesium sulfate administration on blood-brain barrier in a rat model of intraperitoneal sepsis: a randomized controlled experimental study. Crit Care. 2005; 9:R18–23. 10.1186/cc3004 15693962 PMC1065104

[47] Tang XN, Berman AE, Swanson RA, Yenari MA. Digitally quantifying cerebral hemorrhage using photoshop and image J. J Neurosci Methods. 2010; 190:240–43. 10.1016/j.jneumeth.2010.05.004 20452374 PMC2898728

[48] Park DW, Lee IH, Park CW, Seo JT. The effects of vaginal lubricants on the human vagina: an in vitro analysis. Clin Exp Obstet Gynecol. 2019; 46:427–433. 10.12891/ceog4671.2019

[49] Lopes-Rodrigues V, Xavier C, Sousa D, Osório H, Assaraf YG, Lima RT, Vasconcelos H. ALIX protein analysis: storage temperature may impair results. J Mol Clin Med. 2019; 2:29–34. 10.31083/j.jmcm.2019.02.7161

[50] Chen J, Liu N, Wang X, Zhao Y, He J, Yang L, Sun Q, Zhao J, Wang L, Chen L. Dl-3-n-butylphthalide inhibits phenytoin-induced neuronal apoptosis in rat hippocampus and cerebellum. J Integr Neurosci. 2019; 18:277–83. 10.31083/j.jin.2019.03.174 31601076

[51] Li ZF, Liu HB, Li JG, Lang JH, Zhang GR He ZX. Changes in Ovarian Function Induced by Letrozole in an Endometriosis Rat Model. J Reprod Med. 2019; 64:21–27.

[52] Tas ZA, Ozgur T, Yaldiz M, Dolapcioglu K. MMP7 and YKL-40 expression in endometrial endometrioid carcinomas. Eur J Gynaecol Oncol. 2019; 40:123–129. 10.12892/ejgo4515.2019

