# Supplementary material for: L-3-n-butylphthalide attenuates inflammation response and brain edema in rat intracerebral hemorrhage model
Source: Aging (Albany NY). 2020 Jun 21;12(12):11768–80. doi: 10.18632/aging.103342 (PMC7343495; doi:10.18632/aging.103342)
Supplement: Supplementary Figure 1 [file aging-12-103342-s001..pdf]

SUPPLEMENTARY FIGURE

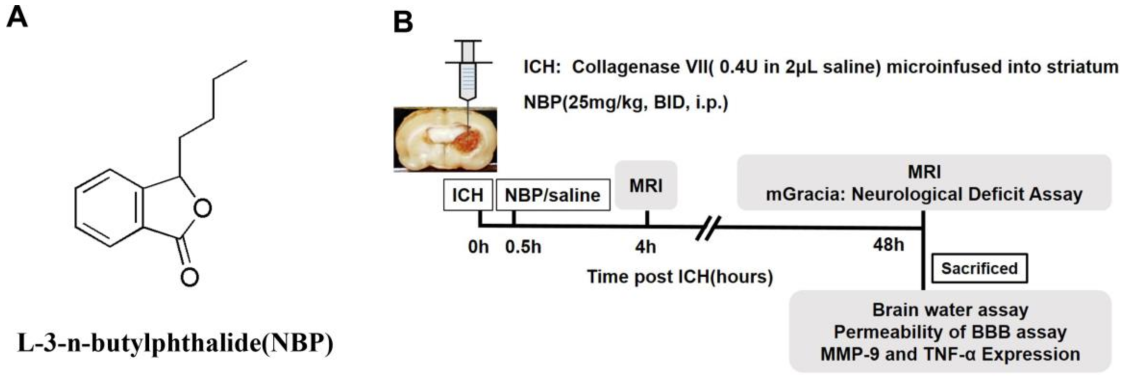

Supplementary Figure 1. The chemical structure of NBP and the experimental design. (**A**) The chemical structure of NBP; (**B**) The schematic diagram of experimental design. ICH: intracerebral hemorrhage; BID: twice-daily; i.p.: intraperitoneal; NBP: butylphthalide; MMP-9: matrix metalloproteinase-9; TNF-α: tumor necrosis factor-alpha; BBB: blood-brain barrier; MRI: magnetic resonance imaging.
